# Wafer-Bonded AlGaInP Red LEDs with Suppressed S‑Droop through Surface Sulfidation

**DOI:** 10.1021/acsami.5c20576

**Published:** 2026-01-30

**Authors:** Je-Sung Lee, Seung-Hyun Mun, Sunwoo Shin, Rae-Young Kim, Seung Hyeok Lee, Sugyeong Cha, Hye-Sung Han, Kyung-Pil Kim, Hoe-Min Kwak, Jaeyoung Baik, Soo-Young Choi, Sang-Jo Kim, Woo-Lim Jeong, Jun-Youn Kim, Sung-Chan Jo, Chang-Mo Kang, Dong-Seon Lee

**Affiliations:** † Department of Electrical Engineering and Computer Science, Gwangju Institute of Science and Technology (GIST), 123 Cheomdangwagi-ro, Buk-gu, Gwangju 61005, Republic of Korea; ‡ Department of Semiconductor Engineering, Gwangju Institute of Science and Technology (GIST), 123 Cheomdangwagi-ro, Buk-gu, Gwangju 61005, Republic of Korea; § Samsung Display Co. Ltd., 1 Samsung-ro, Giheung-gu, Yongin-si, Gyeonggi-do 17113, Republic of Korea; ∥ Department of Nanomechatronics Engineering, 34996 Pusan National University, Geumjeong gu,Busan 46241, Republic of Korea

**Keywords:** micro LED, LEDoS, size-effect, S-droop, red LED, AlGaInP, passivation, ammonium sulfide

## Abstract

Micro–light-emitting diode (micro-LED) technology enables high pixels-per-inch (PPI) displays using conventional semiconductor processes and relies on extremely miniaturized mesa structures derived from traditional LEDs. Scaling to smaller sizes leads to a significant decrease in emission efficiency because of the stronger influence of sidewall damage. This efficiency degradation, known as the size-effect or S-droop, primarily arises from surface defects introduced by dry etching. These defects promote nonradiative Shockley–Read–Hall (SRH) recombination and create pathways for surface leakage current. In aluminum gallium indium phosphide (AlGaInP) LEDs, chemical passivation with ammonium sulfide is widely used to mitigate sidewall damage. However, the underlying reaction mechanism remains unclear, and most studies address only the sidewall regions. In this work, we fabricated a wafer-bonded vertical AlGaInP LED structure compatible with light-emitting diode on silicon (LEDoS) integration and applied ammonium sulfide surface sulfidation to investigate its broader effects. Chemical changes from sulfidation were examined using energy dispersive spectroscopy (EDS) and X-ray photoelectron spectroscopy (XPS). These analyses revealed that surface defects were replaced by stable sulfur bridge bonds, leading to Fermi level unpinning. Electrical characterization further separated parallel and series resistances, which revealed the influence of surface sulfidation across both the top and sidewall interfaces. As a result, the maximum external quantum efficiency (EQE) increased by 120.6% at 5 A/cm^2^ in a 10 μm pixel, accompanied by a clear reduction in S-droop.

## Graphical abstract

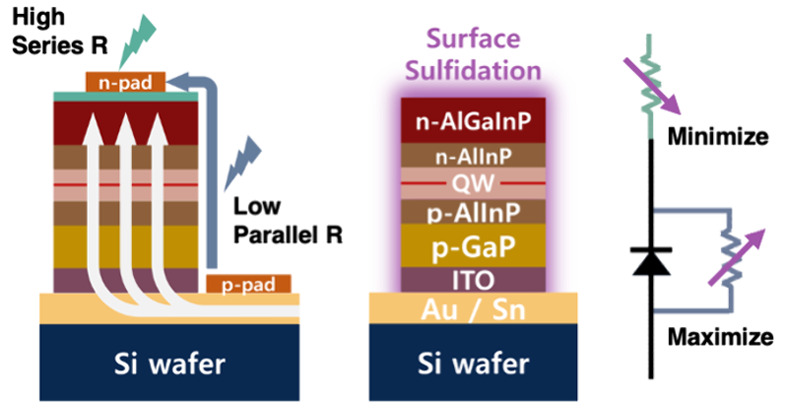

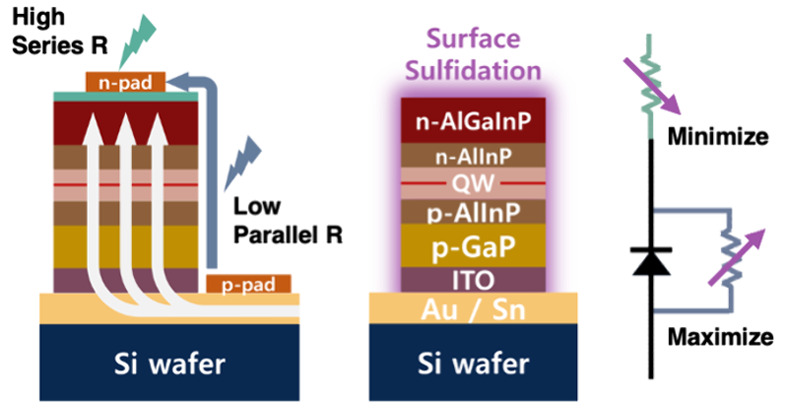

## 1 Introduction

Micro–light-emitting diodes (micro-LEDs) have emerged as strong candidates for next-generation displays such as smart glasses, head-up displays, and extended reality (XR) systems.^1–3^ Their inorganic nature ensures compatibility with advanced semiconductor fabrication processes and enables the integration of ultrahigh pixel-per-inch (PPI) displays.^4,5^ In addition, micro-LEDs provide unmatched brightness, vivid color purity, extended operational lifetimes, and excellent environmental stability—key attributes that distinguish them from conventional OLED technologies.^1,3^

Micro-LED display pixels consist of red, green, and blue subpixels, each requiring semiconductors with distinct band gaps.^5,6^ While blue and green emissions are efficiently realized using indium gallium nitride (InGaN)-based LEDs,^7^ both aluminum gallium indium phosphide (AlGaInP)- and InGaN-based LEDs are under active investigation for red emission.^8^ InGaN-based red LEDs, which share the same material system as blue and green devices, offer strong chemical and thermal stability.^9^ However, they suffer from relatively low efficiency and poor color purity in the red spectral region and show a blue shift under high current injection because of the quantum-confined Stark effect (QCSE).^10,11^ Their most common substrate, sapphire, is electrically insulating, which necessitates complex integration processes such as laser lift-off (LLO) or chemical lift-off (CLO).^12,13^ In contrast, AlGaInP-based red LEDs achieve high efficiency and excellent color purity and are typically grown on conductive gallium arsenide (GaAs) substrates, which allow straightforward substrate removal via wet etching.^7,14^ Despite these advantages, AlGaInP faces several challenges, including chemical instability from its multicomponent metallic composition and limited post process compatibility due to its low growth temperature.^15,16^ A particularly critical issue is the sharp drop in emission efficiency during pixel miniaturization, commonly referred to as the size effect or S-droop.^17,18^ This degradation is worsened by the inherently high surface recombination velocity (SRV) of AlGaInP, which makes the material especially sensitive to surface damage introduced during device fabrication.^17,19^

S-droop, observed in both InGaN and AlGaInP LEDs, is mainly attributed to increased SRH recombination and enhanced surface leakage current caused by damage during mesa etching.^17,20^ In particular, inductively coupled plasma reactive ion etching (ICP-RIE) can lead to plasma-induced sidewall defects and introduce various forms of surface contamination, including polymeric and metallic species.^19,21^ As pixel size decreases, the edge-to-area ratio (E/A) increases, which magnifies the effect of such damage.^18,22^ To address this problem, alternative methods such as wet etching,^2,23^ neutral ion beam etching,^24^ and etching-free passivation have been explored,^25,26^ although their material specificity and limited scalability restrict practical use. As a result, many studies focus on postfabrication treatments, particularly slight sidewall etching followed by passivation.^19,27,28^ Wet chemical treatments—such as KOH, TMAH, HF, and ammonium sulfide—have proven effective in reducing S-droop by removing plasma-induced damage.^27,29,30^ In addition, dielectric coatings such as Al_2_O_3_, SiO_2_, and SiN_*x*_, deposited via plasma-enhanced chemical vapor deposition (PECVD), sputtering, or atomic layer deposition (ALD), as well as solution-based methods such as octadecylthiol (ODT) or sol–gel, have also been investigated.^19,31–33^

However, direct deposition of dielectric layers (e.g., via ALD or PECVD) onto plasma-damaged surfaces presents a critical challenge for AlGaInP devices. Unlike InGaN, where wet etchants like KOH effectively remove damage prior to passivation,^34^ AlGaInP lacks compatible etchants.^28^ Consequently, physical encapsulation alone entraps surface defects and residues, failing to effectively suppress interface recombination.^35^ Therefore, ammonium sulfide treatment is indispensable as it uniquely provides a ‘slight etching’ effect to clean the surface prior to passivation, in addition to its chemical passivating role.

In this context, surface sulfidation with ammonium sulfide remains virtually the only viable approach for achieving damage-free passivation in AlGaInP LEDs.^36^ This is mainly due to the high chemical reactivity (i.e., high electropositivity) of aluminum (Al), which makes it difficult to achieve uniform and controllable slight etching.^15^ Ammonium sulfide has been used for oxide removal and passivation in GaAs devices for a long time, and several studies have extended this method to AlGaInP LEDs.^36,37^ However, some of these reports examined only photoluminescence (PL) enhancement without fabricating devices, while others presented electroluminescence (EL) results from ammonium sulfide-treated LEDs but observed only modest performance improvements.^19,28^ Comprehensive investigations of the surface chemical transformations and oxide-removal behavior induced by ammonium sulfide remain limited.^19,28^

In this study, we present a detailed analysis of surface sulfidation in wafer-bonded vertical AlGaInP LEDs using ammonium sulfide, focusing on the correlation between surface chemical modifications to improvements in electrical and optical performance. Systematic characterization demonstrates substantial efficiency enhancement and effective suppression of S-droop, particularly in microscale pixel structures.

## 2 Results and Discussion

### 2.1 Structural and Electrical Impact of Surface Sulfidation in Vertical Micro-LEDs

Unlike InGaN-based LEDs, which require complex lift-off processes, AlGaInP-based red LEDs enable selective substrate removal via wet etching and allow for straightforward vertical micro-LED formation through low-temperature eutectic bonding.^38^ This wafer-level bonding facilitates integration with complementary metal-oxide-semiconductor (CMOS) driver circuits, making it well-suited for LED-on-Silicon (LEDoS) technology and high-PPI XR microdisplays.^38^ Based on this structure, we fabricated a eutectic-bonded vertical AlGaInP micro-LED – see Figure 1a. The process began with the deposition of indium tin oxide (ITO) on the red epitaxial wafer and an Au/Sn bonding layer on both the epi and Si substrates. After eutectic bonding under heat and pressure, the GaAs substrate and etch-stop layer (n-GaInP) were removed via acid–based wet etching. Mesa pixel structures were then defined by dry etching, followed by surface sulfidation with aqueous ammonium sulfide and metal pad deposition to complete the device. To investigate how this sulfidation process influences the final device performance, we analyzed the fabricated structure and modeled its equivalent circuit, as shown in Figure 1b. This model illustrates the primary current conduction path in the LED and identifies two critical parasitic components: *R*_p_ and *R*_s_.

**Figure 1:**
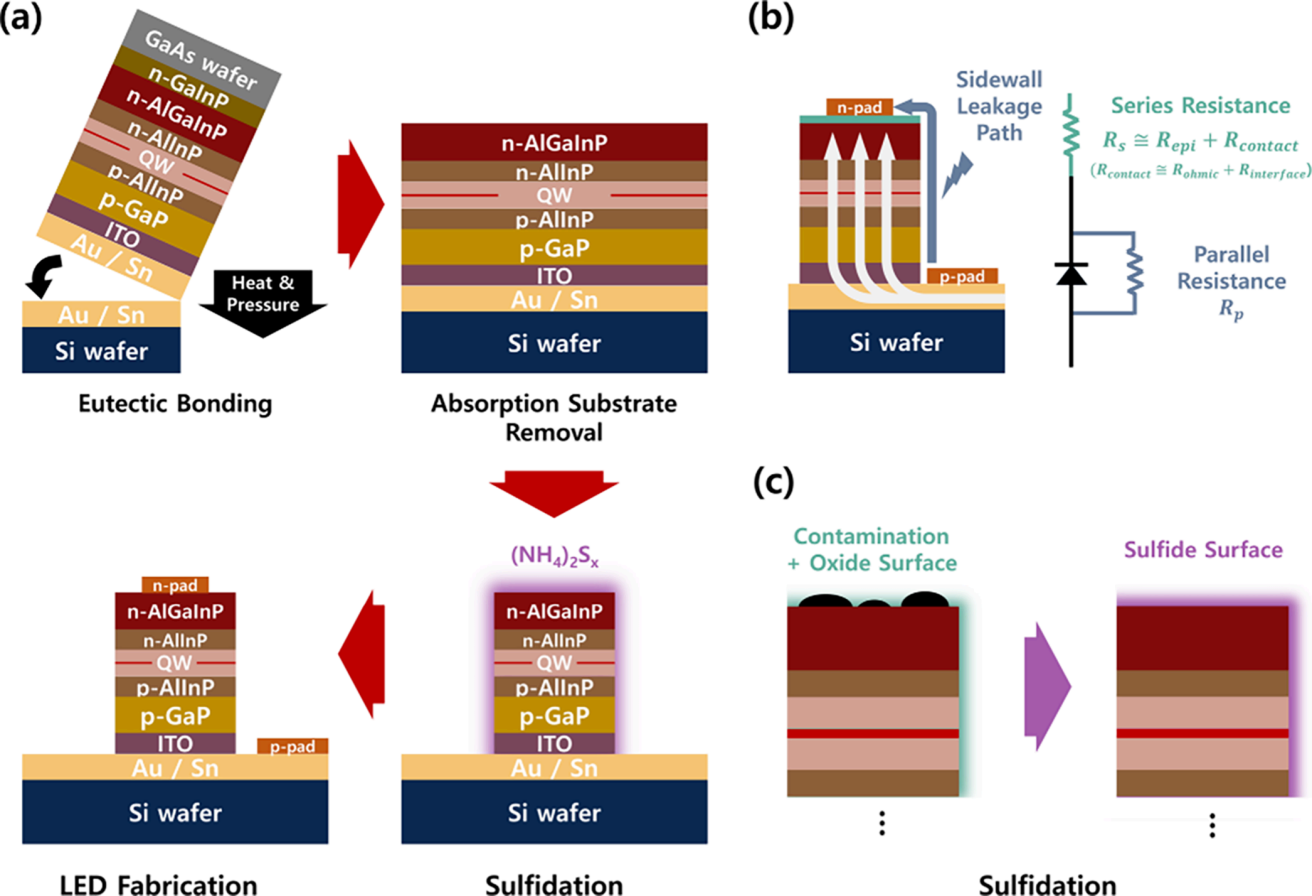
(a) Schematic illustration of the wafer-bonded AlGaInP red LED fabrication process. (b) Equivalent circuit model of the LED structure, featuring parallel resistance (*R*_p_) for surface and bulk leakage paths and series resistance (*R*_s_) for transport-related voltage drop. (c) Conceptual illustration of the surface sulfidation effect, showing the transformation from an oxidized surface to a stabilized sulfide layer.

The majority of the carriers injected between the n- and p-type pads undergo radiative recombination within the quantum wells (QWs), as indicated by the light gray arrows. However, a portion of the carriers bypass the QWs via leakage routes along the mesa surface, known as sidewall leakage paths (blue-gray path).^35^ These leakage paths act as a *R*_p_ (blue-gray resistor), which is the first parasitic element originating from sidewalls that are structurally unstable and vulnerable to environmental exposure. Such instability leads to the formation of native oxides, moisture absorption, and surface contamination, all of which introduce defect states.^17,39,40^ Furthermore, during dry etching, high-density plasma can induce ion bombardment, generating additional trap states originating from etching species.^17^ The leakage current, diverted from the injected current before reaching the active region, reduces both the internal quantum efficiency (IQE) and the current injection efficiency (CIE).^35^

In addition to *R*_p_, the second parasitic element, *R*_s_ (teal-gray resistor), causes voltage drop and thermal accumulation, both of which further degrade the overall device efficiency.^38,41^ *R*_s_ comprises epitaxial resistance (*R*_epi_), which is defined by the intrinsic LED structure, and contact resistance (*R*_contact_), determined by the metal–semiconductor interface, as illustrated in Figure 1b. *R*_contact_ includes ohmic resistance (*R*_ohmic_) and interfacial resistance (*R*_interface_), the latter caused by native oxides or contaminants that impede carrier injection.^42^ Whereas *R*_epi_ is largely fixed during epitaxial growth, *R*_contact_ depends on subsequent processing. Elevated *R*_s_ can hinder carrier transport, reduce CIE, and increase heat generation, which ultimately degrades EQE.^41^

As both parasitic resistances can significantly degrade performance, a surface treatment strategy capable of addressing both *R*_p_ and *R*_s_ is essential. Hence, we employed surface sulfidation with ammonium sulfide. Ammonium sulfide has been widely used to remove native oxides from III–V semiconductors, effectively replacing oxide layers with sulfides.^30,36^ Typically, combining slight surface etching with dielectric deposition eliminates trapped ions and plasma-induced damage while passivating residual dangling bonds with deposited films.^19,28^ Plasma treatments^43^ or single/multilayer dielectrics^44^ without etching have shown some EQE improvement, but they remain limited in eliminating inherent surface defects.^30,45^ Slight etching is particularly problematic for AlGaInP LEDs. For example, Jung et al. showed that diluted HF acid successfully removed sidewall damage, highlighting its potential as a surface treatment method, though care must be taken to avoid excessive etching of the AlInP cladding layer.^15^ These results highlight the difficulty of using acid or alkaline etchants for AlGaInP. In contrast, ammonium sulfide does not cause excessive etching and has been shown to improve LED efficiency when followed by dielectric deposition.^19,28^ It therefore serves as a mild etchant that removes native oxides and contaminants, including polymeric and metallic residues, without structural damage. Furthermore, as shown in Figure 1c, ammonium sulfide can be applied simultaneously to both the mesa sidewalls and the top contact surface, which makes it a practical solution for comprehensive surface treatment. Our experimental design was based on this dual applicability. To validate the effectiveness of this surface sulfidation approach across varying device dimensions, we designed four types of mesa structures with different edge lengths, areas, and calculated E/A ratios as summarized in Table 1.

**Table 1:** Key Parameters Used in the Experimental Model

mesa size	edge length (μm)	area (μm^2^)	E/A
10 × 10 μm^2^	40	100	0.4
25 × 25 μm^2^	100	625	0.16
50 × 50 μm^2^	200	2500	0.08
100 × 100 μm^2^	400	10,000	0.04

To minimize confounding factors in the analysis of *R*_p_, partial sidewall insulation layers for top contact routing were intentionally omitted, and a direct contact configuration was employed instead. For the smallest 10 × 10 μm^2^ chips, precise probing was achieved with a fine probe tip of 1 μm diameter, which allowed direct contact with the top surface. For all chip sizes, the metal pad was designed to cover 20% of the chip area, which ensured a consistent emission area ratio across devices.

### 2.2 Sulfidation of Sidewall Defects for Enhanced Parallel Resistance (*R*_p_)

As described above, ammonium sulfide enables simultaneous treatment of both the top and sidewall surfaces, allowing control over *R*_s_ and *R*_p_, respectively. To examine its chemical effect on the sidewall, we employed annular dark-field scanning transmission electron microscopy (ADF-STEM) combined with energy dispersive spectroscopy (EDS). For precise observation of the active region, a representative MQWs epitaxial structure was used, and mesa structures were fabricated following the same process as the devices. A cross-sectional STEM image of the resulting mesa is shown in Figure 2a. As illustrated in the inset, the mesa extends from the GaAs substrate through the AlGaInP window layer, AlInP cladding layer, MQWs, and up to the p-GaP window layer. The STEM image confirms that the sidewall was cleanly defined by dry etching, with no noticeable roughness or recession. The MQWs region marked by the white dashed box in Figure 2a was magnified for EDS mapping. Figure 2b shows the results for the reference sample without sulfidation, while Figure 2c shows the sulfide-treated sample. In both cases, the ion-overlapped image (top left) and the Ga mapping (top right) clearly reveal the sidewall boundary of the MQWs. The most pronounced difference in surface chemistry between the two samples is observed in the bottom-left oxygen ion and bottom-right sulfur ion mapping images. In the reference (Figure 2b), a distinct oxide layer (green) is uniformly distributed along the sidewall, while sulfur signals are sparse and randomly distributed.

**Figure 2:**
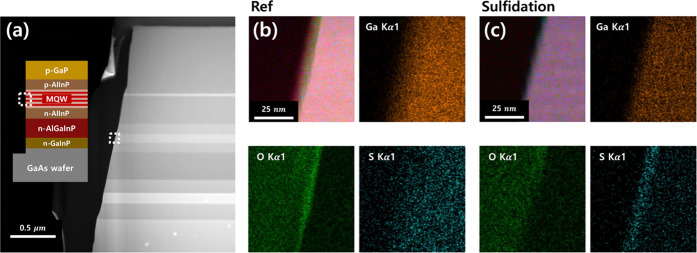
(a) Cross-sectional ADF-STEM image of the AlGaInP LED mesa structure, accompanied by a schematic diagram. The white dotted box marks the region analyzed by EDS near the multiquantum wells (MQWs). (b, c) EDS mapping images showing the distributions of overlapped elements Ga, O, and S for (b) the reference and (c) the sulfide-treated mesa sidewalls.

In contrast, the sulfide-treated sample (Figure 2c) exhibits no discernible oxide layer. Instead, a thin sulfur-rich layer (highlighted in cyan) appears uniformly distributed along the sidewall. These results provide experimental validation of previous reports that the native oxide layer on III–V semiconductor surfaces can be effectively replaced by a sulfide layer through sulfidation.^30,36^

To complement the STEM and EDS mapping results, which confirmed the removal of native oxides and the formation of a sulfide layer on the mesa sidewall, we conducted a more quantitative, chemical-state-specific analysis using XPS. To maximize sidewall exposure within the X-ray spot, we fabricated a square mesa array on an epitaxial wafer containing MQWs, as illustrated in Figure 3a. Each mesa was 10 × 10 μm^2^ in size with a 10 μm spacing and was formed using the same dry etching process as in the actual devices. Given the elliptical X-ray spot (∼400 μm in major axis), the measurement covered more than 50 mesa sidewalls, ensuring statistically meaningful surface-averaged data.

**Figure 3:**
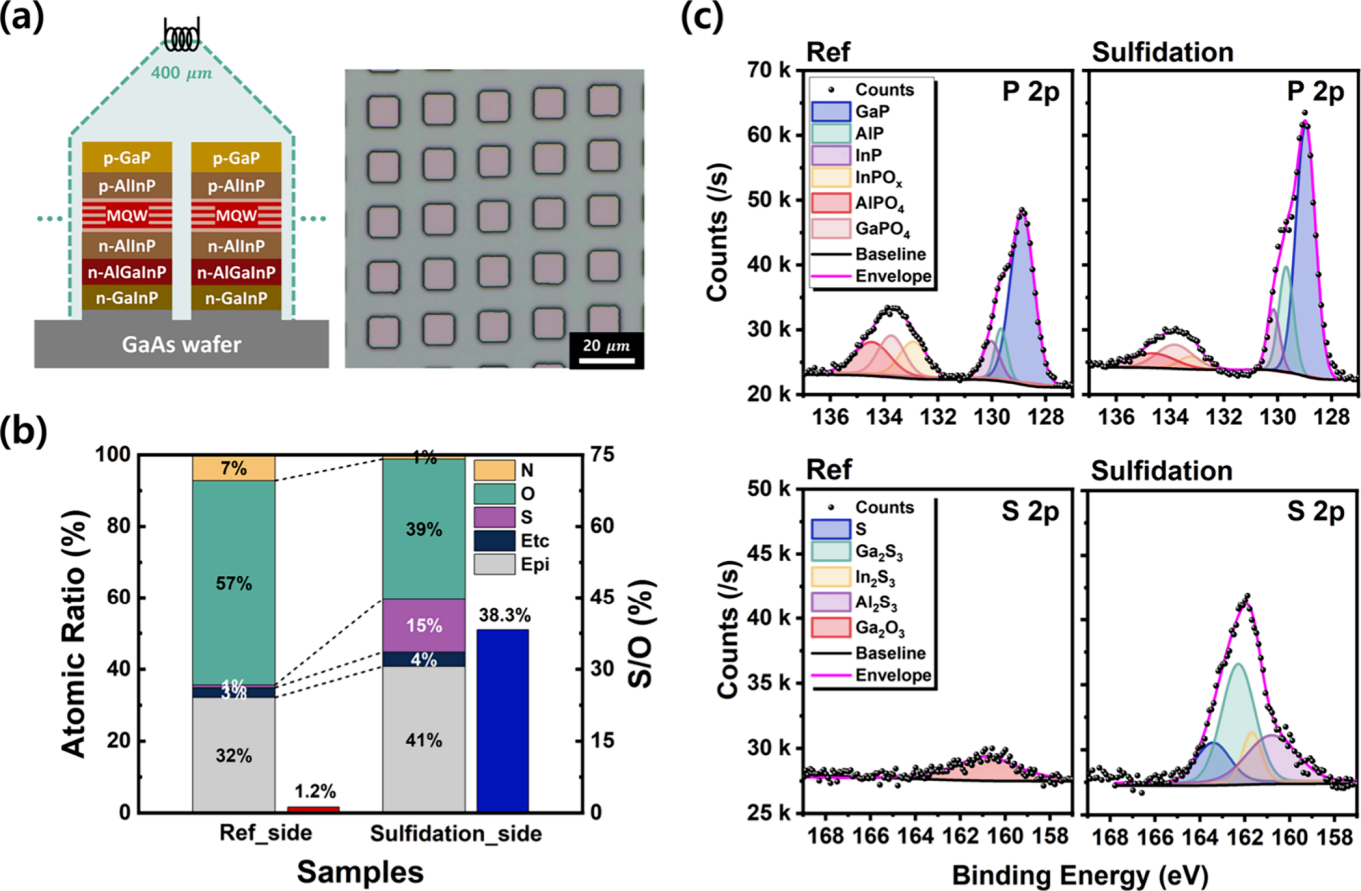
(a) Schematic of the mesa array structure used for X-ray photoelectron spectroscopy (XPS) analysis (left), and the corresponding optical microscope (OM) image of the XPS sample (right). (b) Surface atomic composition and sulfur-to-oxygen (S/O) ratio for the reference and sulfide-treated samples, as determined by XPS. (c) High-resolution XPS spectra of the P 2p and S 2p core levels, with peak fitting for both untreated and sulfidated mesa arrays.

Figure 3b shows the relative atomic ratios extracted from the XPS spectra. The detected elements were grouped into nitrogen, oxygen, sulfur, and “Epi”, which includes Ga, Al, In, and P from the epitaxial layers. The most notable effect of sulfidation was a reduction in oxygen content from 57 to 39%, accompanied by an increase in sulfur from 1 to ∼15%. This result quantitatively supports the oxide-to-sulfide substitution observed in Figure 2. The S/O ratio further confirms this change, increasing from 1.2 to 38.3% after sulfidation. A slight decrease in nitrogen indicates the removal of adsorbed nitrogen species. The apparent increase in the “Epi” and “Etc” categories arises from the relative reduction in oxygen, rather than true elemental enrichment.

Figure 3c shows the deconvoluted P 2p and S 2p spectra. For P 2p, peaks were fitted using reference binding energies for GaP (129.2 eV),^46^ AlP (129.6 eV),^47^ InP (130.0 eV),^48^ InPO_4_ (133.6 eV),^49^ AlPO_4_ (133.7 eV),^47^ and GaPO_4_ (134.2 eV).^46^ Although a complete fitting using the single AlGaInP material proved challenging, the reference sample clearly exhibited two distinct regions: the Metal phosphide peaks (Al-, Ga-, In–P) in the 128–131 eV range and the surface metal phosphates peaks (Al-, Ga-, In-PO_4_) derived from native oxide in the 132–136 eV range.

In the sulfide-treated sample, these oxide-related peaks were significantly suppressed, concurrent with a uniform increase in the intensities of all Metal phosphide peaks (Al-, Ga-, In–P). Quantitatively, the oxide-to-AlGaInP peak ratio decreased sharply from 39.3 to 15.1%, demonstrating a 61.4% reduction in phosphate species. This collectively suggests that all constituent elements of AlGaInP uniformly form a surface oxide layer, which is then effectively removed following the sulfidation process.

The S 2p spectra were fitted using binding energies for Al_2_S_3_ (160.1 eV),^50^ In_2_S_3_ (161.85 eV),^51^ Ga_2_S_3_ (162.2 eV),^52^ and elemental sulfur (163.1 eV).^53^ Because sulfur is not introduced during epitaxial growth, the small peak in the reference sample is attributed to the 3s peak of Ga_2_O_3_ near 160.7 eV.^54^ In contrast, the sulfide-treated sample exhibited strong sulfide peaks, confirming the formation of a sulfide passivation layer with Al, Ga, and In ions.

Plasma etching at the mesa sidewall can result in ion trapping, particularly of chlorine (Cl^–^) species originating from etching gases.^17,29^ In order to assess this effect, Cl 2p core-level spectra were analyzed, as shown in Figure 4. The Cl 2p peak typically appears as a doublet, consisting of the Cl 2p_3/2_ peak in the 198–201 eV range and the Cl 2p_1/2_ peak, separated by approximately 1.6 eV, with an area ratio of 2:1.^55^ Since the separation between these two peaks is narrow, a broad overall peak is observed. Consequently, the Cl 2p_3/2_ and Cl 2p_1/2_ peaks form tails extending to lower or higher energies due to bonding with other elements.^55^ We hypothesized that metal chlorides were formed via bonding with the constituent elements of the epi-layer (Al, Ga, In, and P). Given the highly similar distribution of the relevant peaks, an integrated fitting approach was employed to reduce the potential for subjective interpretation.

**Figure 4:**
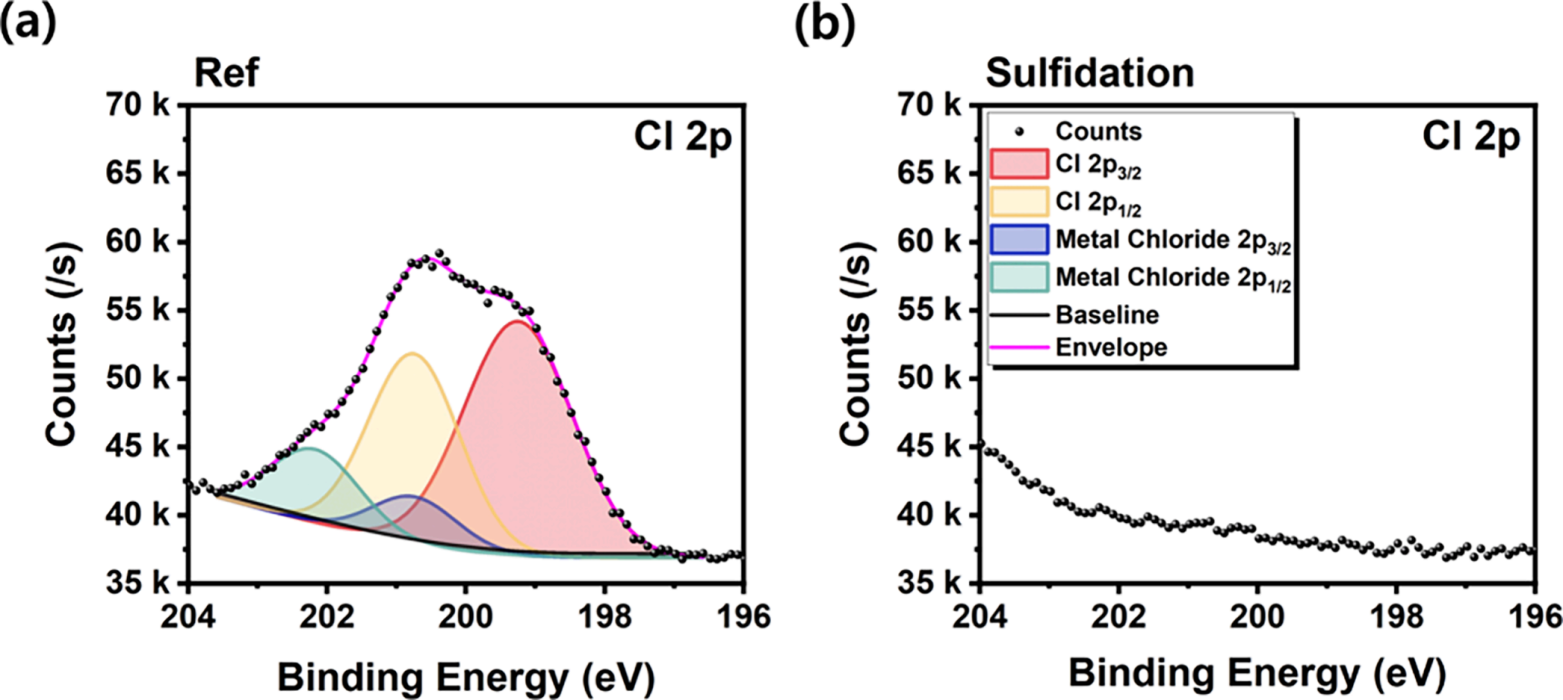
High-resolution XPS spectra of Cl 2p core levels for (a) the reference and (b) the sulfide-treated mesa arrays, with deconvoluted peaks indicating changes in surface chlorine species.

A clear reduction in Cl-related signals after sulfidation indicates that ammonium sulfide treatment helps remove plasma-induced surface contaminants. These results provide direct experimental confirmation of the proposed mechanism: ammonium sulfide performs mild etching of surface defects—including native oxides and trapped ions—while simultaneously forming a uniform sulfide passivation layer. Notably, metal phosphates were replaced by metal sulfides, respectively confirming successful chemical conversion. These findings collectively reaffirm the dual functionality of ammonium sulfide: enabling mild sidewall etching and effective removal of dry-etching-induced contaminants.

### 2.3 Sulfidation of the Top Surface to Reduce Series Resistance (*R*_s_)

To examine the effects of sulfidation on the mesa top surface, XPS was performed on wafer-bonded epitaxial layers from the device structure. After eutectic bonding, the substrate was removed, and dry etching was carried out. Unlike the sidewall-focused measurements, this XPS setup probed only the mesa top surface. As illustrated in Figure 5a, an elliptical X-ray beam with a major axis of 150 μm was positioned to irradiate the top surface exclusively.

**Figure 5:**
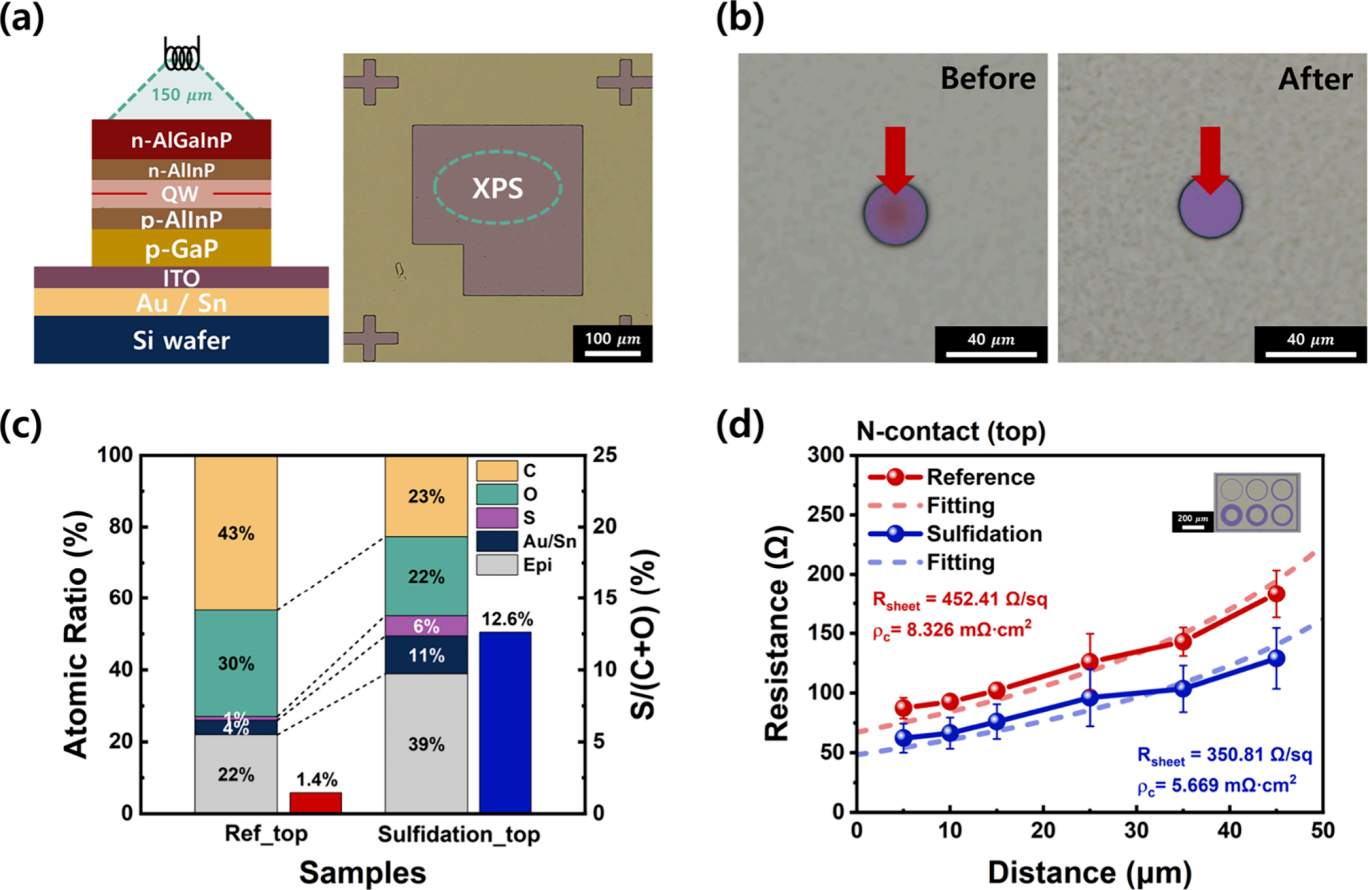
(a) Schematic illustration of the mesa structure used for XPS measurements (left), and OM image of the corresponding sample (right). (b) OM images of the top surface before and after sulfidation, showing removal of photoresist (PR) residues. (c) Atomic composition analysis from XPS and the calculated sulfur-to-(carbon + oxygen) ratio. (d) Resistance measurements using the circular transmission line method (CTLM), with fitted curves used to extract sheet and specific contact resistances for both the reference and sulfide-treated samples. The inset shows the fabricated CTLM structure.

Circular mesa structures were also fabricated to allow visual comparison of surface conditions before and after sulfidation. Figure 5b shows that red PR residues (red arrows) remained on the surface even after stripping, most likely due to high-energy plasma exposure during dry etching. These burned PR contaminants can persist at the metal–semiconductor interface, hindering the formation of ohmic contacts and increasing contact resistance.^56^ Elevated contact resistance increases the overall *R*_s_, which leads to heat accumulation and reduced CIE.^57^ Oxygen plasma ashing can help remove residues, but it may also increase surface oxide content. In contrast, sulfidation was expected to eliminate both native oxides and polymer residues. As shown in the right panel of Figure 5b, the PR residues were completely removed, which confirms that sulfidation provides effective top-surface cleaning.

A detailed evaluation of PR contamination removal was performed using relative atomic ratios from XPS, as shown in Figure 5c. While the previous XPS analysis in Figure 3 focused on oxide removal and sulfur passivation of the sidewall, this analysis tracked changes in polymer-related species (mainly carbon and oxygen) on the mesa top surface.^58^ Figure 5c categorizes the atomic composition into the following: red LED epitaxial elements (“Epi”), gold–tin alloy from the eutectic bonding layer (“Au/Sn”), sulfur for sulfidation analysis, and oxygen and carbon as contamination indicators. Comparing the reference (left) and sulfide-treated (right) samples reveals a decrease in oxygen and an increase in sulfur, which is consistent with the sidewall results. The oxygen content decreased by 8%, while sulfur increased by 5%. This modest change likely reflects the smaller X-ray spot size and the more complex elemental composition introduced by the eutectic bonding layer. Importantly, both carbon and oxygen (key components of polymer residues)^58^ decreased together. Carbon content dropped sharply from 43 to 23%, suggesting substantial PR residue removal. To quantify this effect, the sulfur-to-(carbon + oxygen) ratio increased from 1.4 to 12.6% after sulfidation. Although part of this change reflects native oxide removal, the sharp ratio increase confirms effective polymer elimination. The apparent increases in the Au/Sn and Epi categories are primarily due to the relative decrease in carbon and oxygen, rather than true elemental gains. These results demonstrate that ammonium sulfide removes both native oxides and PR residues, thereby improving the cleanliness of the contact interface.

To distinguish the respective effects of PR residue removal and native oxide etching, XPS was also performed on a wafer-bonded epitaxial sample without mesa etching, as shown in Figure 1a. Because this sample was not exposed to dry etching, no plasma-induced PR burning was expected; thus, surface changes observed after sulfidation can be attributed mainly to native oxide removal. As shown in Figure S1b, which represents a sample without PR coating, the atomic ratios reveal a 6% reduction in carbon, a 15% reduction in oxygen, and a 3% increase in sulfur. Compared to the main device structure (where carbon and oxygen decreased by 20 and 8%, respectively), this indicates that sulfidation effectively removes native oxides when absence of polymer contamination. By contrast, PR-coated samples exhibited more dramatic concurrent reductions in both carbon and oxygen, which shows that sulfidation removes both polymer residues and native oxides simultaneously. Because PR is an oxygen-containing polymer,^58^ the oxygen reduction in these samples likely reflects contributions from both oxide etching and polymer degradation. These results collectively indicate that sulfidation enables simultaneous removal of native oxides and PR through a unified surface reaction mechanism. The fitted P 2p spectra in Figure S1c further show a reduction in metal phosphate species, consistent with the trends observed in the sidewall analysis.

As discussed above, polymer contamination has a critical impact on contact resistance at the semiconductor–metal interface. To directly evaluate this effect, we employed ammonium sulfide sulfidation to remove both native oxides and residual PR from the top surface. The resulting changes in contact resistance were measured using the CTLM.^59^ CTLM patterns were fabricated alongside the LED devices, and resistance values were recorded between top-side n-contact pads with varying gap distances, as shown in Figure 5d. An OM image of the CTLM structure is provided in the inset. To ensure statistical reliability, resistance measurements were performed on six identical devices. The measured values at each gap spacing were averaged, and the resulting data points were fitted using a nonlinear regression model to extract the sheet resistance (*R*_sheet_) and specific contact resistance (ρ_c_), as shown in Figure 5d. The reference sample exhibited values of *R*_sheet_ = 452.41 Ω/sq and ρ_c_ = 8.326 mΩ cm^2^. In contrast, the sulfide-treated sample demonstrated improved electrical characteristics with *R*_sheet_ = 350.81 Ω/sq and ρ_c_ = 5.669 mΩ cm^2^, corresponding to reductions of 22.5 and 31.9%_,_ respectively. These findings align with the surface analyses and demonstrate that ammonium sulfide effectively removes both native oxides and polymer contaminants that would otherwise hinder ohmic contact formation.

To further elucidate the fundamental passivation mechanism governed by these chemical changes, we focus on the bonding configurations at the interface and their impact on the electronic band structure.

First, regarding the physical surface conditions illustrated in Figure 6a, the oxidized AlGaInP surface typically possesses unsaturated dangling bonds and defective native oxides, which serve as active surface states.^45,60^ Concurrently, polymeric and metallic contaminants from prior processing remain on the surface. Our study reveals that the ammonium sulfide treatment induces a “chemical lift-off” effect (Figure 5b). This mechanism is primarily driven by the slight etching of the underlying native oxide layer, which effectively detaches these residues. Furthermore, metallic residues undergo a chemical conversion into metal sulfides, which are either dissolved into the solution or passivated (Figure S3b), thereby eliminating conductive leakage paths.

**Figure 6:**
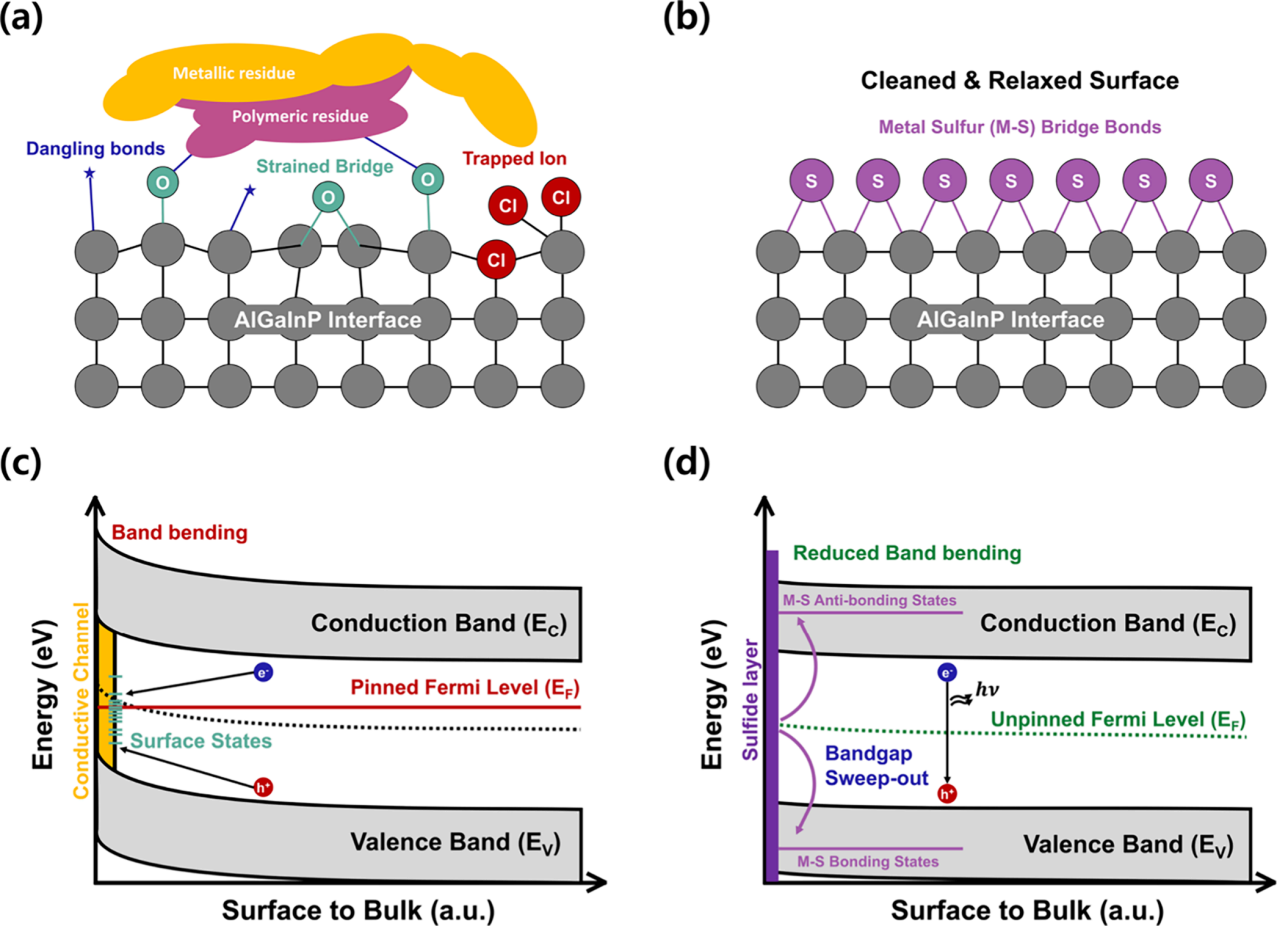
Mechanism of AlGaInP interface restoration via sulfide passivation. (a, b) Schematic comparison of the physical and atomic surface structure before (a) and after (b) sulfidation. Corresponding evolution of the interface energy bandgap diagram before (c) and after (d) sulfidation.

As presented in Figure 6b, upon sulfidation, sulfur (S) atoms effectively replace surface oxygen atoms and occupy vacancies.^60^ Due to its high chemical affinity, sulfur forms stable covalent bonds with group-III metals (Al–S, Ga–S, In–S), typically occupying bridge sites.^45,60^ As validated by our XPS analysis (Figures 3–5), this conversion of metal phosphates to metal sulfides effectively saturates dangling bonds, terminating the surface with a chemically stable sulfide layer. It is noteworthy that this passivation layer functions primarily as a self-limiting chemical termination rather than a thick crystalline epitaxial film.^60^ Therefore, its uniformity and effectiveness are validated by the completeness of chemical bonding and the subsequent suppression of leakage currents, rather than by long-range crystallographic order.

Figure 6c,d schematically illustrate the evolution of the energy band diagram near the QW surface before and after sulfidation, respectively. In the pretreatment state (Figure 6c), unlike an ideal band structure, a high density of surface states is formed within the bandgap due to native oxides and plasma damage. These midgap states facilitate SRH recombination rather than radiative recombination. Furthermore, these surface states induce unwanted Fermi level pinning and severe band bending, resulting in the formation of a depletion region and a potential barrier. This increases *R*_epi_, a component of the *R*_s_ described in Figure 1b, which ultimately leads to reduced EQE and increased heat generation. Additionally, the conductive polymeric and metallic residues on the surface create parasitic conduction channels, leading to a decrease in *R*_p_, which can further degrade EQE. On the top side, such pinning and contamination hinder ohmic contact formation, increasing *R*_c_, which also contributes to the overall increase in *R*_s_.

However, postsulfidation (Figure 6d), the removal of native oxides and contaminants allows sulfur ions to form bridge bonds with surface atoms.^45,60^ This process splits the unstable surface states into bonding states (deep within the valence band) and antibonding states (within or above the conduction band), exhibiting the “bandgap sweep-out” effect.^60,61^ Consequently, Fermi level pinning is resolved, and band bending is restored to a near-flat band condition. With the simultaneous elimination of leakage paths caused by surface contamination, the interface recovers to a near-ideal band structure, thereby significantly improving the device EQE.

### 2.4 Optical Validation of Electrical Improvements from Sulfidation

The surface and electrical analyses described above confirmed that oxide defects and polymer contaminants introduced during mesa etching can be effectively removed by ammonium sulfide treatment. This process suppresses sidewall leakage and surface SRH recombination, thereby improving *R*_p_, while also reducing contact resistance at the top electrode, lowering *R*_s_. To assess the impact on device operation, LED structures were fabricated and evaluated through EL measurements.

J–V characteristics were measured for devices of various mesa sizes. Figure 7a,b show averaged log-scale curves from five reference and five sulfide-treated samples. Both exhibited diode-like behavior, but the reference devices showed noticeable leakage near 0 V. The symmetric leakage under forward and reverse bias suggests surface-related current paths. An inflection near 2 V, corresponding to LED turn-on, was observed for all sizes. This likely marks the point where the diode’s dynamic resistance falls below *R*_p_, diverting most of the current through the active region. At higher bias, *R*_p_ plays a smaller role as diode conduction dominates. In sulfide-treated devices (Figure 7b), reverse current remained near the detection limit. The slight size-dependence is attributed to area normalization across all device sizes.

**Figure 7:**
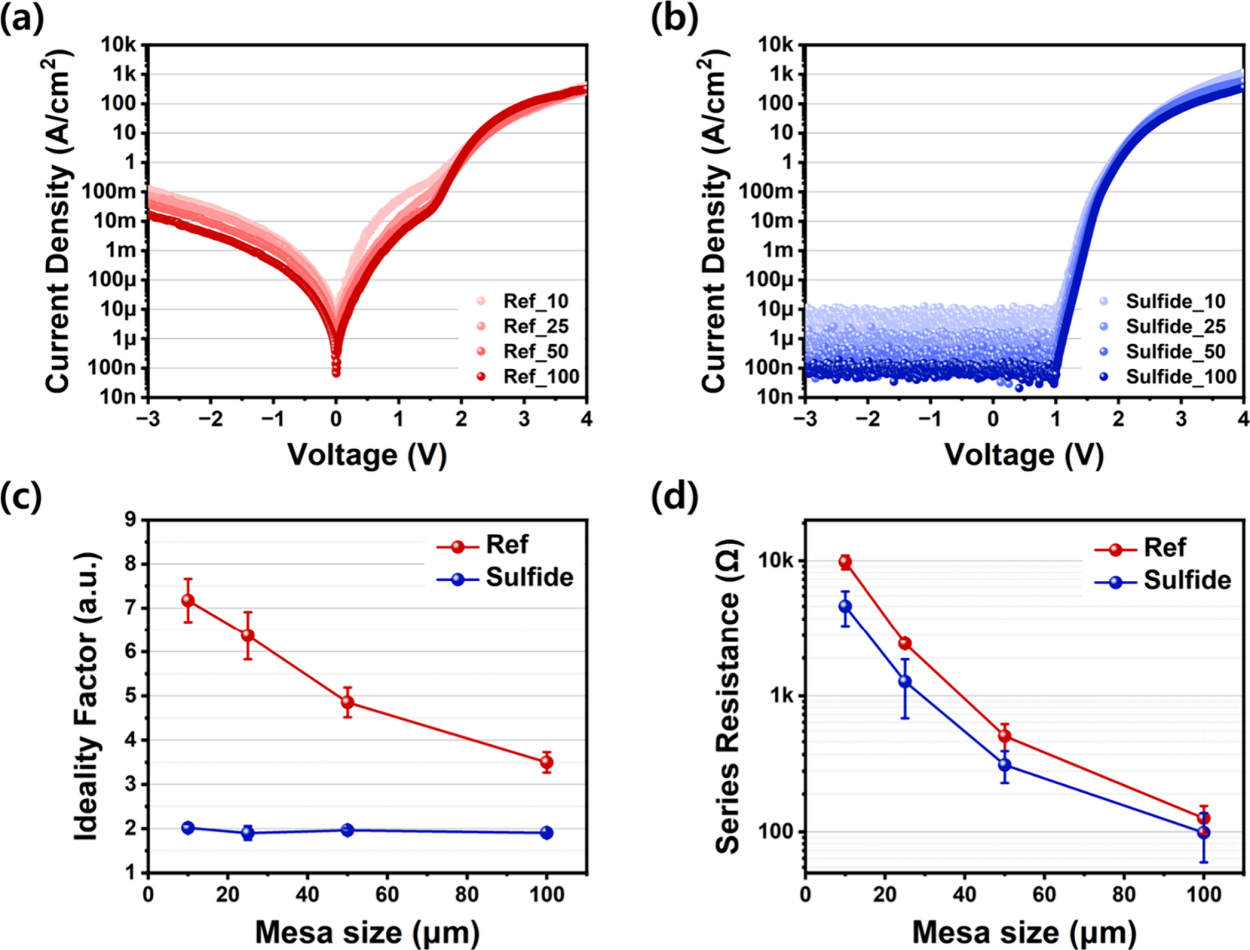
(a, b) Current density–voltage (J–V) characteristics of (a) reference and (b) sulfide-treated LEDs with varying mesa sizes. (c) Extracted ideality factors and (d) calculated *R*_s_ at a forward bias of 4 V. All measurements were performed on LEDs with lateral dimensions ranging from 10 × 10 to 100 × 100 μm^2^.

To further clarify how surface sulfidation influences device behavior, a quantitative analysis was performed to examine the relationship between SRH recombination, surface leakage, and *R*_p_.

Equation 1 Surface SRH recombination rate

$$R_{\mathrm{SRH}}=(A_{\mathrm{bulk}}+A_{\mathrm{surface}})n=A_{\mathrm{bulk}}\cdot n+\left(S\frac{A_{s}}{V}\right)\cdot n$$ 1

SRH recombination, a key contributor to S-droop, lowers *R*_p_ by promoting leakage current during device operation. To better understand surface-related SRH effects, we focus here on the SRH recombination rate (*R*_SRH_), particularly the contribution from surface defects. As shown in eq 1, *R*_SRH_ can be separated into two coefficients (*A*_bulk_ and *A*_surface_) depending on the recombination site. The surface component *A*_surface_ is expressed as the product of SRV (S) and the surface-to-volume ratio (*A*_s_/*V*). Assuming a uniform active-layer thickness, *A*_s_/*V* can be approximated by the edge-to-area ratio (E/A), making *A*_surface_ strongly dependent on mesa size. By contrast, *A*_bulk_, which reflects internal defects such as traps or dislocations, remains largely unaffected by mesa formation. Previous studies^19,62,63^ have inferred that *A*_surface_ increases with decreasing mesa size. Accordingly, the present analysis focuses on *A*_surface_ to isolate surface defect contributions, excluding *A*_bulk_.

Beyond recombination losses, surface defects can also create unintended leakage-current paths, which further decrease the EQE. These leakage paths may arise from surface defects, organic contaminants, native oxides, dielectric layer properties, or ambient moisture.^17,39,40^ Formed along the mesa sidewalls, they divert carriers away from the active region, which lowers *R*_p_. During dielectric deposition—particularly PECVD—the vulnerable sidewalls are further exposed to plasma, potentially creating additional defects.^27^ In some micro-LED structures with lateral p-contact pads, partial insulation layers along the sidewalls can also act as leakage paths, depending on their thickness and quality.^35^

Equation 2 Modified Shockley Equation Incorporating Non–Ideal Effects

$$I_{\mathrm{diode}}=I_{s}(e^{qV/nkT}-1)$$ 2a

$$I=I_{\mathrm{diode}}+(I_{\mathrm{SRH\_surface}}+I_{\mathrm{leakage}})=I_{s}(e^{qV/nkT}-1)+\frac{V}{R_{p}}$$ 2b

To analytically evaluate the impact of SRH recombination and leakage paths on *R*_p_, we applied the Shockley diode equation, as shown in eq 2a. Here, *I*_diode_ denotes the ideal diode current, *I*_s_ is the reverse-bias saturation current, *q* is the elementary charge, *V* is the applied voltage, *n* is the ideality factor, *k* is the Boltzmann constant (1.38 × 10^–23^ J/K), and *T* is the absolute temperature. Equation 2a represents the nonleakage model where *R*_p_ approaches infinity. In real devices, however, deviations occur, as shown in eq 2b, which includes additional current terms from surface SRH recombination (*I*_SRH_surface_) and leakage (*I*_leakage_). These nonideal currents can be collectively represented by the voltage-dependent term *V*/*R*_p_, which reflects finite *R*_p_. As discussed earlier, removing sidewall defects through targeted surface treatments suppresses both surface SRH recombination and parasitic leakage. Consequently, this increases *R*_p_ and brings the current–voltage characteristics closer to the ideal case. Because the S-droop effect becomes more pronounced with decreasing pixel size (primarily due to stronger sidewall influence), these results suggest that effective surface passivation is a promising strategy to mitigate efficiency losses in miniaturized devices.

Building on the previous discussion, numerical values of *R*_p_ were extracted from the J–V characteristics of actual LEDs for comparison. Although *R*_p_ was estimated simply using *R* = *V*/*I* at −1 V reverse bias (a simple comparative indicator),^64^ a more accurate determination would require fitting the full I–V curve to an equivalent circuit model.^65^ Figure S2a shows the extracted values for both device types. As expected, *R*_p_ decreased with increasing mesa size. Sulfide-treated samples exhibited values approximately 10^3^× higher than the reference, with less variation across sizes. Based on the standard deviation, no statistically significant size dependence was observed, suggesting strong suppression of surface-driven leakage by sulfidation.

To further investigate surface SRH effects, *R*_p_ values were compared under −1 and +1 V bias (Figure S2b). Under reverse bias, resistance increased with decreasing mesa size, which is consistent with geometry-related leakage paths. However, under forward bias, the trend was weaker, which suggests an asymmetric response. This asymmetry suggests that forward-injected carriers undergo nonradiative recombination at sidewall traps near the QWs, which increases leakage and lowers resistance.^66^ In contrast, reverse leakage is dominated by tunneling or trap-assisted generation.^66^ Sulfide-treated devices maintained stable resistance across sizes and biases, supporting the conclusion that sulfidation suppresses both surface leakage and SRH-related recombination.

Equation 3 Ideality factor in the low–injection regime:

$$n=\frac{q}{kT}\cdot \frac{dV}{d(\ln\;I)}$$ 3

To assess sidewall-induced nonidealities, the ideality factor (*n*) was extracted from low-injection I–V data using eq 3. This parameter, derived from the Shockley equation, reflects recombination and defect-related effects. Typically, *n* ≈ 1 indicates ideal diode behavior, *n* ≈ 2 suggests that recombination processes (including SRH and radiative recombination) dominate, and *n* > 2 reflects nonidealities such as surface traps, leakage, a degraded *R*_p_.^67^

Figure 6c shows extracted n values for current densities below 10 A/cm^2^. In reference devices, n increased from 3.50 (100 μm) to 7.17 (10 μm), demonstrating strong nonideality due to sidewall leakage and SRH recombination. This size-dependent trend reflects the impact of the increasing E/A. In contrast, sulfide-treated devices maintained nearly constant *n* (1.9–2.0), indicating effective surface passivation. Although not ideal, this behavior suggests that bulk SRH recombination remains the dominant mechanism.

We now focus on the changes induced by top-surface sulfidation. Sulfidation improves this region by removing polymer and oxide contaminants, thereby lowering contact resistance, as confirmed by CTLM. This reduces overall *R*_s_, which is particularly important under high current, where it limits conduction and degrades EQE. Figure 7d presents the estimated *R*_s_ measured at +4 V, averaged over ten devices for each size to ensure statistical reliability. Both device types showed increased resistance with decreasing size, consistent with the inverse relationship between resistance and current-path area. At 10 μm, the reference device exhibited (9.79 ± 1.13) kΩ, while the sulfide-treated device showed (4.61 ± 1.34) kΩ—a 52.9% reduction. This confirms that sulfidation improves the top-contact interface and enhances EQE during high-current operation.

To evaluate how improved electrical properties affect optical performance, EL analysis was conducted, with all results representing the average of five measured devices to ensure reliability. Figure 8a shows emission patterns of 10 and 100 μm devices at 0.1 and 0.5 A/cm^2^. The 10 μm reference device showed no emission at 0.1 A/cm^2^ due to leakage through low *R*_p_, while the sulfide-treated device exhibited faint but visible emission, confirmed by intensity contours. At 0.5 A/cm^2^, the treated device produced strong, uniform emission, while the reference remained weak. In the 100 μm devices, both samples emitted at 0.1 A/cm^2^, but the sulfide-treated device was significantly brighter. That the 10 μm device failed to emit while the 100 μm device did, clearly illustrates the more pronounced S-droop phenomenon in smaller devices. This contrast highlights the stronger S-droop effect in smaller devices. By comparison, sulfide-treated devices consistently emitted across all sizes and current densities. Figure 8b presents EL spectra at 10 A/cm^2^, where all devices showed similar peak wavelengths (636.07 ± 0.34 nm), but sulfide-treated samples exhibited higher intensity.

**Figure 8:**
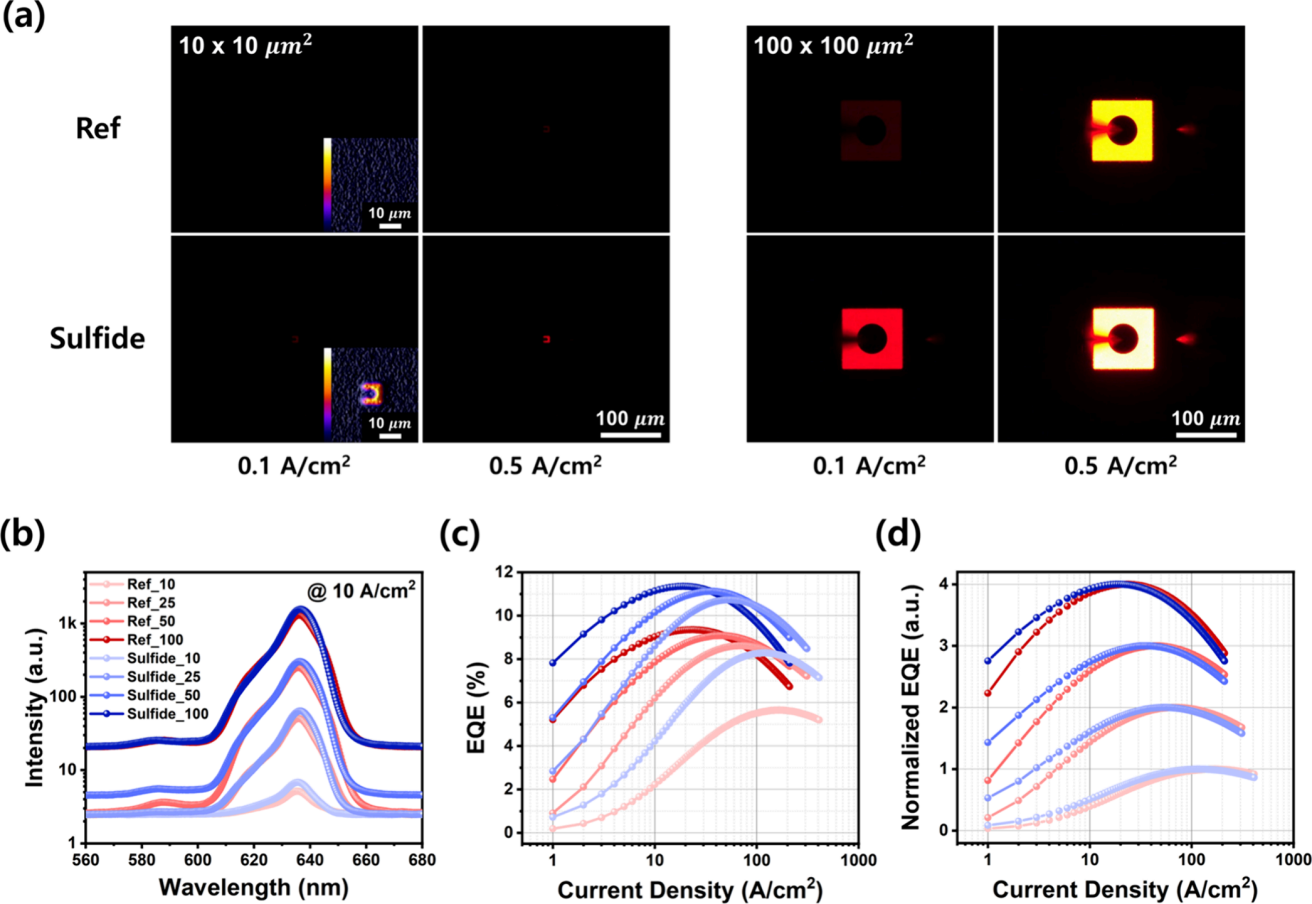
(a) EL images of reference and sulfide-treated LEDs with lateral dimensions of 10 × 10 and 100 × 100 μm^2^, measured at 0.1 and 0.5 A/cm^2^. Insets in the 10 μm images show contour maps of the light intensity distribution. (b) Emission spectra on a logarithmic scale. (c) EQE and (d) normalized EQE as a function of current density, with curves vertically shifted for clarity. All data correspond to reference and sulfide-treated LEDs with mesa sizes from 10 × 10 to 100 × 100 μm^2^.

To further probe radiative efficiency, EQE was calculated from optical power measured with top/bottom integrating spheres under on-wafer conditions– see Figure 8c. The 50 μm reference device reached 6.6% EQE, an improvement over the 2.8% previously reported for similar LEDs,^44^ likely due to GaAs substrate removal in wafer-bonding.

EQE typically decreased as device size decreased. At 5 and 100 A/cm^2^, the reference group exhibited EQE reductions of 85.5 and 32.8%, respectively, while the sulfide-treated group showed smaller drops of 74.6 and 14.6%. These results agree with the electrical analyses: increased *R*_p_ improved performance at low current densities, while reduced *R*_s_ enhanced performance at high current densities. Together, these effects suppressed size-dependent degradation across all device dimensions.

Sulfidation yielded significant EQE gains across sizes. At 5 A/cm^2^, EQE increased by 26.5, 36.8, 53.6, and 120.6% for the 100, 50, 20, and 10 μm devices, respectively. At 100 A/cm^2^, the increases were 17.9, 19.6, 22.4, and 49.8%. The 10 μm device, with the highest E/A ratio, showed a striking 120.6% improvement at low current—one of the highest values reported for AlGaInP LED passivation. These data strongly support that sulfidation not only recovers etched sidewalls but also provides effective sulfur passivation, which suppresses both surface SRH recombination and leakage pathways.

S-droop was assessed by comparing EQE peak reductions between 100 and 10 μm devices: 39.5% for the reference and 27.0% for the sulfide-treated group. Although full size-independence was not achieved (likely due to residual resistance, SRH recombination, etching damage, probe shading, and measurement limits), the reduced S-droop indicates effective suppression of surface-related losses. To visualize this, weighted normalized EQE curves were plotted in Figure 8d.

At low current densities, where *R*_s_ is negligible, EQE is governed mainly by *R*_p_ through its influence on CIE and IQE. At high current densities, *R*_s_, carrier overflow, and Auger recombination degrade EQE. This trade-off produces a peak, which shifts depending on the dominant factor. While previous studies have shown that strong SRH recombination shifts EQE peaks to higher current densities,^19^ our postsulfidation results revealed a consistent shift toward lower current densities across all sizes. Specifically, the peak shifted from 23 to 19 A/cm^2^ (−17.4%) in the 100 μm device and from 168 to 121 A/cm^2^ (−28.0%) in the 10 μm device. These shifts emphasize the role of sulfidation, particularly in small devices with higher E/A ratios. Analysis of EQE gains further revealed that improvements were generally larger at low current than at high current, which suggests a stronger influence from sidewall recovery than from top-contact optimization. In summary, while sulfidation passivates both top and sidewall surfaces, its dominant effect arises from sidewall recovery, which explains the leftward shift in EQE peak positions.

### 2.5 Long-Term Stability and Aging Characteristics

While the immediate performance enhancement is evident, ensuring long-term reliability is equally critical for practical display applications. The proposed sulfidation process operates through two primary mechanisms: the slight etching of fabrication-induced damage (including native oxides as well as polymeric and metallic contaminants) and the substitution of dangling bonds with sulfides. As demonstrated, these mechanisms effectively eliminate leakage pathways and suppress surface SRH recombination.

However, a key challenge remains: the sulfide layer formed by this process is intrinsically vulnerable to oxidation and hydrolysis when directly exposed to ambient air and humidity.^68^ Consequently, while the “slight etching” effect provides durable leakage suppression by removing physical defects, the “chemical passivation” effect may degrade over time. Therefore, to evaluate the environmental stability of the sulfidation treatment, we conducted a time-aging experiment on the fabricated devices to track variations in their electrical and optical characteristics.

Figure 9 compares the initial measurement results (from Section 2.4) with the remeasured results for sulfide-treated samples stored for 6 months at room temperature in ambient air. Representative device sizes of 10 × 10 and 100 × 100 μm^2^ were used, and all data points represent the average of 5 measured devices.

**Figure 9:**
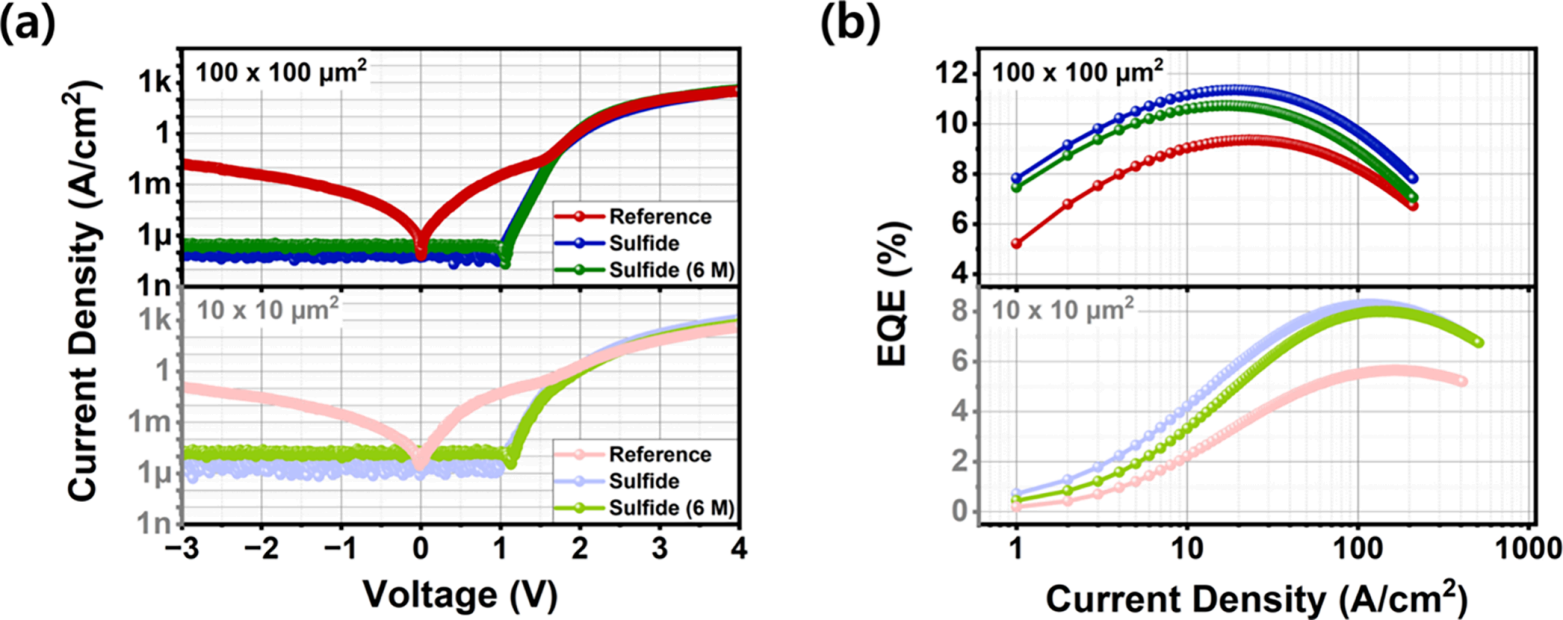
(a) Current density–voltage (J–V) characteristics and (b) external quantum efficiency (EQE) of reference, sulfide-treated, and 6-month aged sulfide sample (6 M). The results are presented for devices with lateral dimensions of (Upper) 10 × 10 μm^2^ and (Below) 100 × 100 μm^2^.

In Figure 9a, the average reverse current density at −3 V for the 10 μm devices was recorded as (1.21 ± 0.82) × 10^–1^, (1.87 ± 2.24) × 10^–6^, and (12.82 ± 2.27) × 10^–6^ A/cm^2^ for the reference, sulfide, and aged sulfide samples, respectively. Similarly, the 100 μm devices showed (1.69 ± 0.57) × 10^–2^, (1.11 ± 0.56) × 10^–7^, and (3.19 ± 1.15) × 10^–7^ A/cm^2^. The sulfide and aged sulfide devices exhibited analysis results within the error range of the measurement equipment at ∼ pA level, while the reference maintained the ∼ μA level leakage current. The fact that leakage current did not reappear in the 6-month-aged devices strongly suggests that the primary leakage pathways, which were due to contaminants and damage removed by the slight etching mechanism, were effectively eliminated and remain suppressed. This proves the durability of the leakage current suppression, even if the sulfide layer’s stability has slightly degraded, potentially increasing SRH recombination.

Next, the EQE analysis presented in Figure 9b reveals a slight difference in efficiency due to aging. The maximum EQE improvement for the 10 μm initial sample was 46.4% compared to the reference, but this decreased slightly to 41.4% for the aged sample. Similarly, the 100 μm device improvement decreased from 21.4 to 14.7%. Even considering measurement error, this indicates a slight decrease over time, which can be attributed to the partial oxidation of the sulfide layer, triggering an increase in SRH recombination. Specifically, the shift of the EQE peak (observed prominently in the smaller size) toward the high current density region is deemed significant (though still well below the reference levels) and suggests increased SRH activity compared to the initial sulfide-treated state, consistent with the provided previous reports.^68^

In summary, the first effect of sulfidation (slight etching) maximizes efficiency improvement by removing the primary cause of leakage pathways, and this effect remains durable over time. However, while the second effect (sulfide layer formation) is critical for maximizing initial device EQE, it can undergo gradual degradation (e.g., oxidation) upon exposure to ambient air and humidity, leading to increased SRH recombination and reduced efficiency. Consequently, incorporating an additional physical passivation layer (such as a dielectric film) following sulfidation can further maximize the device performance, serving as a robust dual-passivation scheme that secures exceptional long-term stability.

## 3 Methodology

### 3.1 Eutectic Wafer Bonding and Substrate Removal

A GaInP-based red LED epitaxial structure with an optimized thin-film design was grown on a GaAs substrate by metal–organic chemical vapor deposition (MOCVD). The structure consisted of an n-GaInP etch-stop layer, an n-AlGaInP window layer, an n-AlInP cladding layer, an AlGaInP/GaInP single quantum well (SQW), a p-AlInP cladding layer, and a p-GaP window layer. An indium tin oxide (ITO, 250 nm) layer was deposited by sputtering on the p-GaP to form the p-ohmic contact, followed by rapid thermal annealing (RTA) at 300 °C for 5 min under nitrogen.

For eutectic bonding, Au/Sn/Au (150/150/20 nm) was deposited on the LED epi by e-beam evaporation. On the Si target substrate, Ti/Ni/Au (50/50/300 nm) was used as an adhesion layer. The wafers were bonded face-to-face at 320 °C under 1200 kgf for 30 min. After cooling, the bonded sample was immersed in a 1:1 ammonium hydroxide/hydrogen peroxide solution at 50 °C and stirred until the GaAs substrate was removed, which exposed the etch-stop layer. A subsequent 1 min dip in 1:1 hydrochloric acid/deionized (DI) water removed the etch-stop layer. The sample was then rinsed, dried, and diced for LED processing.

### 3.2 Fabrication of Sulfidation-Treated Vertical Micro-LEDs

Mesa structures were defined on the wafer-bonded epi using AZ-4330 positive PR (MicroChem) and photolithography, followed by BCl_3_-based ICP-RIE down to the Au/Sn bonding layer. Residual PR was removed with acetone.

For surface sulfidation, samples were immersed in ammonium sulfide solution ((NH_4_)_2_S, 40–48 wt % in H_2_O; Sigma-Aldrich) for 30 min at room temperature to treat both the exposed sidewalls and mesa tops. The entire process was conducted in a fume hood with controlled temperature and humidity, and samples were subsequently stored in a nitrogen (N_2_) desiccator to minimize exposure to ambient humidity and oxygen. Detailed experimental results regarding the optimization of the treatment duration are provided in Figure S3 of the Supporting Information. After DI and IPA rinsing, metal contact pads were defined using AZ-2035 negative PR (MicroChem). N-type contacts (Pd/Ge/Au, 50/50/200 nm) and p-type contacts (Cr/Au, 30/300 nm) were deposited by e-beam evaporation and patterned by lift-off. A second RTA step at 250 °C for 30 min in nitrogen was performed to improve ohmic contact.

### 3.3 Structural, Chemical, and Optical Characterization

To investigate sulfur substitution of native oxides on the sidewalls, an MQWs epi structure with a wide active region was fabricated into mesas using the same process as the main devices. After sulfidation, reference and treated samples were prepared for STEM analysis using a focused ion beam system (Hitachi NX5000). High-resolution STEM imaging and EDS mapping were performed with a double Cs-corrected TEM (JEOL JEM-ARM300F2).

For surface chemical analysis, MQWs and SQW samples were patterned into 10 and 300 μm mesa arrays and treated with sulfidation. Binding energy spectra were obtained by XPS (Thermo Fisher Scientific NEXSA). Optical images were acquired with a digital microscope (Olympus DSX1000).

Specific contact resistance was extracted from CTLM analysis using resistance values measured with a source measure unit (Keithley 2400). Electrical and optical performance (including EL imaging) was evaluated with an EL system (Etamax Co.) consisting of a Keithley 2450 SMU, a Newport 2936-R power meter, a Flame–S-UV–vis-ES spectrometer (Ocean Optics), upper and lower integrating spheres, and a glass chuck. Emission contour plots were generated with ImageJ software.

## 4 Conclusions

Wafer bonding offers excellent compatibility with silicon complementary metal-oxide-semiconductor (CMOS) integration and closely matches the fabrication architecture of high-pixels-per-inch (PPI) light-emitting diode on silicon (LEDoS) microdisplays. Based on this approach, we fabricated vertical AlGaInP red LEDs with high color purity. To investigate the S-droop phenomenon and evaluate the effects of surface treatment, LED subpixels with mesa sizes of 10, 25, 50, and 100 μm were implemented. To further enhance external quantum efficiency (EQE) and suppress S-droop, a surface sulfidation process using ammonium sulfide was developed, enabling simultaneous passivation of both the mesa top and sidewall surfaces.

Sidewall sulfidation was expected to mildly etch native oxides, remove plasma-induced etch damage along with polymeric and metallic residues, and passivate exposed dangling bonds with sulfide species. scanning transmission electron microscopy- energy dispersive spectroscopy (STEM-EDS) and X-ray photoelectron spectroscopy (XPS) analyses confirmed oxide-to-sulfide substitution, along with reductions in trapped etching ions and metal phosphates. This passivation suppressed surface Shockley–Read–Hall (SRH) recombination and leakage paths, which increases parallel resistance (*R*_p_).

As a result, leakage current was reduced and ideality factors were stabilized across device sizes. On the top surface, removal of native oxides and polymer contaminants lowered contact resistance, which reduces series resistance (*R*_s_). On-wafer EQE measurements further validated these improvements. For the 10 μm device, a 120.6% increase in EQE at low current density (5 A/cm^2^) was attributed to higher *R*_p_, while a 49.8% enhancement at high current density (100 A/cm^2^) was linked to reduced *R*_s_. After sulfidation, the EQE peak of the 10 μm device shifted 28.0% toward lower current density, which confirms that surface sulfidation mitigates SRH recombination.

Although S-droop was not completely eliminated—likely due to increasing *R*_s_ with size reduction, residual deep etch damage, and measurement limitations—the proposed sulfidation process produced a substantial impact. Given the highly reactive and multicomponent nature of AlGaInP and the difficulty of identifying compatible wet etchants, direct physical encapsulation alone risks entrapping surface defects. In this regard, ammonium sulfide treatment is notable not merely as a standalone method but as an essential surface reconstruction step. Consequently, combining this sulfidation with deposition-based techniques, such as atomic layer deposition (ALD), creates a synergistic dual-passivation scheme. This approach addresses the intrinsic environmental instability of sulfide layers while maximizing interface quality, offering a promising pathway to overcome the S-droop bottleneck in micro-LED display commercialization.

## Supplementary Material



## Notes

Schematic diagram of atomic composition and P 2p spectrum of wafer-bonded samples; extracted parallel resistance values of LEDs; and variation in PL intensity following different sulfidation conditions (PDF)

## ⊥

Daegu Mechatronics & Materials Institute, 32, Seongseogongdan-ro 11-gil, Dalseo-gu, Daegu 42714, Republic of Korea

## #

Electronics and Telecommunications Research Institute, 218 Gajeong-ro, Yuseong-gu, Daejeon 34129, Republic of Korea
