# Supplementary material for: Wafer-Bonded AlGaInP Red LEDs with Suppressed S‑Droop through Surface Sulfidation
Source: ACS Appl Mater Interfaces. 2026 Jan 30;18(5):9054–69. doi: 10.1021/acsami.5c20576 (PMC12903108; doi:10.1021/acsami.5c20576)
Supplement: Supplementary file 1 [file am5c20576_si_001.pdf]

Supporting Information:

Wafer-Bonded AlGaInP Red LEDs with Suppressed S-Droop through Surface Sulfidation

Je-Sung Lee[1], Seung-Hyun Mun[1], Sunwoo Shin[1], Rae-Young Kim[1], Seung Hyeok Lee[1], Sugyeong Cha[2], Hye-Sung Han[1], Kyung-Pil Kim[1,5], Hoe-Min Kwak[1,6], Jaeyoung Baik[1], Soo-Young Choi[1], Sang-Jo Kim[3], Woo-Lim Jeong[3], Jun-Youn Kim[3], Sung-Chan Jo[3], Chang-Mo Kang[4], and Dong-Seon Lee[1,2]**

[1] Department of Electrical Engineering and Computer Science, Gwangju Institute of Science and Technology (GIST), 123 Cheomdangwagi-ro, Buk-gu, Gwangju 61005, Republic of Korea

[2] Department of Semiconductor Engineering, Gwangju Institute of Science and Technology (GIST), 123 Cheomdangwagi-ro, Buk-gu, Gwangju 61005, Republic of Korea

[3] Samsung Display Co. Ltd., 1 Samsung-ro, Giheung-gu, Yongin-si, Gyeonggi-do, Republic of Korea 17113

[4] Department of Nanomechatronics Engineering, Pusan National University, Geumjeong-gu, Busan 46241, Republic of Korea

[5] Present address: Daegu Mechatronics & Materials Institute, 32, Seongseogongdan-ro 11-gil, Dalseo-gu, Daegu, 42714 Republic of Korea

[6] Present address: Electronics and Telecommunications Research Institute, 218 Gajeong-ro, Yuseong-gu, Daejeon, 34129 Republic of Korea

*E-mail: dslee66@gist.ac.kr Tel.: +82-62-715-2248. Fax: +82-62-715-2204.

*E-mail: fd1kcm@pusan.ac.kr Tel.: +82-51-510-6128. Fax: +82-51-514-2358.

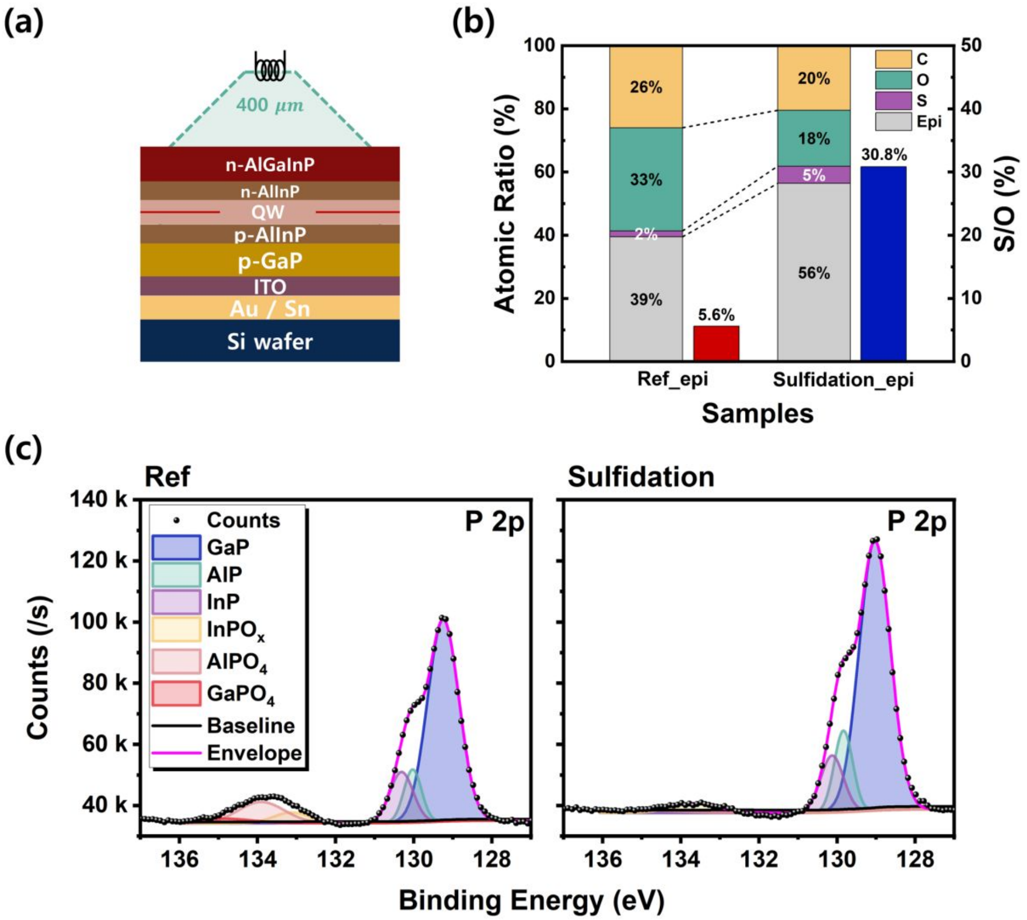

Figure S1. (a) Schematic illustration of the wafer-bonded epitaxial structure used for XPS to evaluate native oxide removal and sulfidation effects without mesa etching. (b) Atomic composition analysis derived from XPS data, highlighting changes in carbon, oxygen, and sulfur contents. Sulfur-to-oxygen (S/O) ratios are also indicated. (c) High-resolution XPS spectra of the P 2p core levels, showing peak deconvolution for both the reference and sulfide-treated samples, with a clear reduction in metal phosphate components after sulfidation.

In order to isolate the effects of native oxide etching from photoresist (PR) residue removal, sulfidation was performed on a wafer-bonded epitaxial sample that had not been exposed to PR.

As illustrated in Figure S1(a), the top surface was analyzed by XPS using a 400 μm X-ray beam, with no mesa patterning required. Figure S1(b) shows atomic ratio data indicating a decrease in oxygen and a corresponding increase in sulfur, which is consistent with native oxide removal and sulfur passivation. The S/O ratio increased from 5.6% in the reference to 30.8% after sulfidation. Although the change was less dramatic than in the sidewall analysis, it confirms that similar chemical processes occur. In Figure S1(c), P 2p spectra were deconvoluted using reference binding energies for GaP (129.2 eV),[1] AlP (129.6 eV),[2] InP (130 eV),[3] $InPO_4$ (133.6 eV),[4] $AlPO_4$ (133.7 eV),[2] and $GaPO_4$ (134.2 eV).[1] The oxide-to-GaInP peak count ratio decreased from 12.5% to 3.3%, which corresponds to a 73.8% reduction in metal phosphate content. This relatively stronger removal effect likely reflects the lower initial oxide content on the top surface, which suggests that sidewall regions are more prone to oxide defect formation after plasma etching.

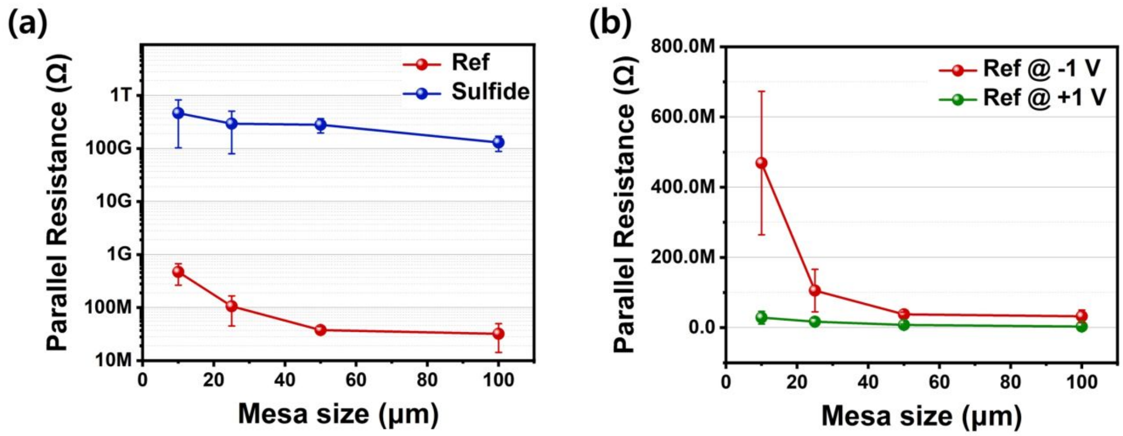

Figure S2. (a) Logarithmic plot of the extracted parallel resistance (R_p) values for reference and sulfide-treated samples at a reverse bias of −1%V, calculated using Ohm's law. (b) Comparison of R_p extracted under reverse (−1%V) and forward (+1%V) bias conditions. All measurements were performed on devices with varying mesa sizes.

To compare the parallel resistance (R_p) of reference and sulfide-treated devices, resistance at −1%V reverse bias was estimated using a simplified Ohm's law approach.[5] For the sulfide-treated [5]samples, smoothing was applied to account for noise near the instrument's detection limit. The extracted values were plotted on a logarithmic scale as a function of mesa size in Figure S2(a), with averages and standard deviations shown as error bars. To further examine the polarity dependence and the influence of surface SRH recombination, R_p values at ±1%V were analyzed for the reference devices, where leakage current was more clearly observed.[6,7] The results are presented in Figure S2(b) on a linear scale. A pronounced asymmetry was observed for smaller device sizes, which indicates stronger surface SRH contributions under forward bias.

(a)

(b)

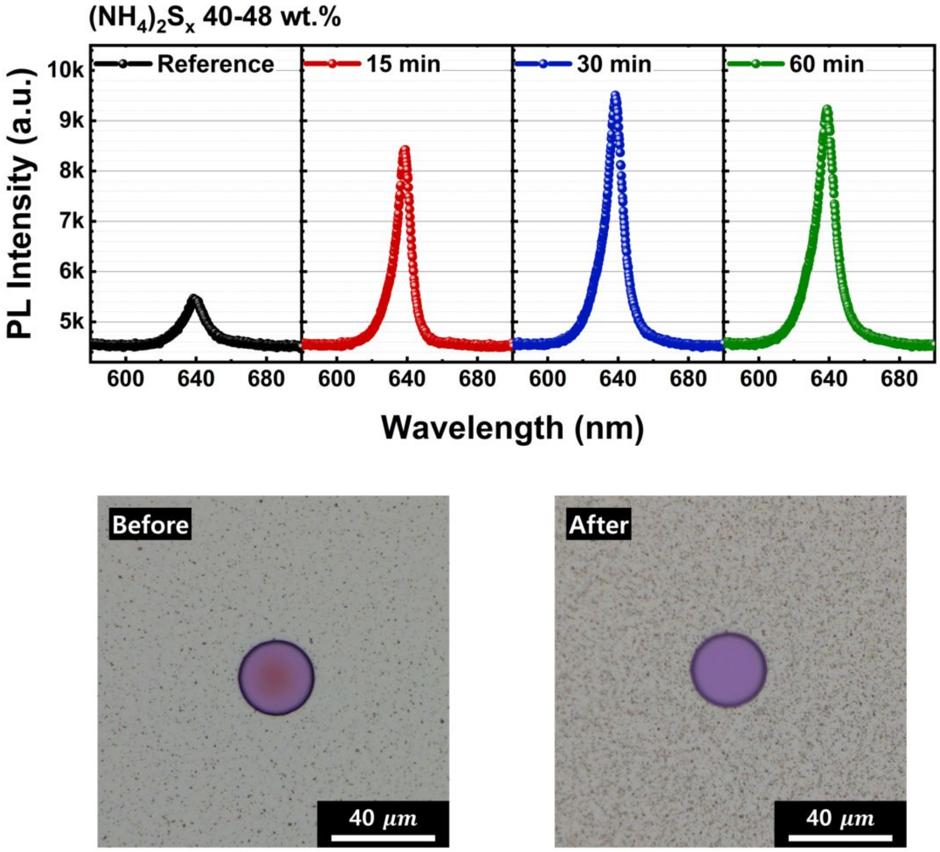

Figure S3. (a) Variation in PL intensity of wafer-bonded LED mesas treated in a 40% aqueous solution with varying treatment durations. (b) Optical microscope (OM) images of the wafer-bonded LED mesa before and after the 30-minute treatment.

To determine the optimized sulfidation procedure, photoluminescence (PL) comparison experiments were conducted.

First, the experiments were limited to room temperature. This decision was driven by practical constraints; specifically, the strong, pungent odor and potential volatility of the ammonium sulfide solution pose significant safety and handling challenges when heated.

Second, the concentration was fixed at the commercially available 40–48 wt.% range, consistent with the solution employed in the main experiments.

Third, the treatment time was optimized, yielding the results presented in Figure S3(a). We observed a progressive increase in PL intensity relative to the untreated device as the immersion time increased up to 30 minutes. However, extending the treatment beyond this point to approximately 60 minutes caused a slight decline in PL intensity. While various factors could contribute to this observation, this result served as a critical indicator of an upper limit for the treatment duration.

Furthermore, the structural specificities of our device—specifically the eutectic Au/Sn bonding layer—played a decisive role in determining the optimal time. While ammonium sulfide solution typically does not directly dissolve bulk metals, it facilitates their separation and removal through the formation of metal sulfide.[8] In our specific material system, while gold (Au) exhibits low reactivity, tin (Sn) is significantly more susceptible to sulfidation. Indeed, Figure S3(b)—which captures the substrate surface of the same device presented in Figure 4(b) of the main text—reveals a noticeable change in surface roughness following the treatment.

This observation confirms a dual effect: while the treatment effectively removes unwanted polymeric and metallic contaminants from the mesa top-side and sidewalls, it also chemically interacts with the exposed regions of the eutectic bonding layer. Although the 30-minute treatment resulted in only minor surface roughness changes, a clear trade-off was identified: extending the duration further could compromise the structural integrity of the bonding layer, thereby degrading

overall device stability. Consequently, the 30-minute duration was selected as the optimal condition to maximize passivation and cleaning effects without inducing significant instability in the device structure.

Collectively, these data provided a sufficient basis to derive the optimal treatment time specific to our wafer-bonded device.

REFERENCES

(1) Franke, R.; Chassé, Th.; Streubel, P.; Meisel, A. Auger Parameters and Relaxation Energies of Phosphorus in Solid Compounds. J. Electron Spectrosc. Relat. Phenom. 1991, 56 (4), 381–388. https://doi.org/10.1016/0368-2048(91)85035-R.

(2) Pan, C.; Li, W.; Jiang, S. Study on the XPS-ESCA of Aluminum Phosphide Products.

(3) Nelson, A. J.; Frigo, S.; Rosenberg, R. Soft X-Ray Photoemission Characterization of the H2S Exposed Surface of p -InP. J. Appl. Phys. 1992, 71 (12), 6086–6089. https://doi.org/10.1063/1.350415.

(4) Zemek, J.; Baschenko, O. A.; Tyzykhov, M. A. Non-Destructive Concentration Depth Profiling of Native-Oxide/InP(100) Samples by Angle-Resolved x-Ray Induced Photoelectron Spectroscopy: Effect of Annealing. Thin Solid Films 1993, 224 (2), 141–147. https://doi.org/10.1016/0040-6090(93)90424-N.

(5) Thompson, P.; Larason, T. Method of Measuring Shunt Resistance of Photodiodes; 2001 Measurement Science Conference, Undefined, 2001.

(6) Lee, M.; Lee, H. U.; Song, K. M.; Kim, J. Significant Improvement of Reverse Leakage Current Characteristics of Si-Based Homoepitaxial InGaN/GaN Blue Light Emitting Diodes. Sci. Rep. 2019, 9 (1), 970. https://doi.org/10.1038/s41598-019-38664-x.

(7) Lee, I.-H.; Kim, T.-H.; Polyakov, A. Y.; Chernykh, A. V.; Skorikov, M. L.; Yakimov, E. B.; Alexanyan, L. A.; Shchemerov, I. V.; Vasilev, A. A.; Pearton, S. J. Degradation by Sidewall

Recombination Centers in GaN Blue Micro-LEDs at Diameters<30 Μm. J. Alloys Compd. 2022, 921, 166072. https://doi.org/10.1016/j.jallcom.2022.166072.

(8) Balakrishnan, A.; Groeneveld, J. D.; Pokhrel, S.; Mädler, L. Metal Sulfide Nanoparticles: Precursor Chemistry. Chem. – Eur. J. 2021, 27 (21), 6390–6406. https://doi.org/10.1002/chem.202004952.
